# Topographical Variation of Iron‐Rimmed Lesions in the Multiple Sclerosis Brain and Spinal Cord: A Neuropathological Study

**DOI:** 10.1002/ana.78113

**Published:** 2026-01-18

**Authors:** Marco Pisa, Andrew Lockhart, Thomas Angell, Aimee Avery, Zaenab Dhari, Mary Bailey, Simon Hametner, Hal Drakesmith, Monika Hofer, Clara Limbaeck, Gabriele C DeLuca

**Affiliations:** ^1^ Nuffield Department of Clinical Neurosciences University of Oxford Oxford UK; ^2^ Mandell MS Center, Trinity Health of New England Hartford CT USA; ^3^ Frank Netter MD School of Medicine Quinnipiac University North Haven CT USA; ^4^ Department of Neuroimmunology Medical University of Vienna Vienna Austria; ^5^ MRC Translational Immune Discovery Unit, MRC Weather all Institute of Molecular Medicine, Radcliffe Department of Medicine University of Oxford Oxford UK; ^6^ Neuropathology, Oxford University Hospital NHS Foundation Trust Oxford UK

## Abstract

Paramagnetic‐rim lesions are a novel diagnostic marker in multiple sclerosis (MS) and are associated with poor prognosis due to their link with chronic inflammation and disease progression. Analyzing 46 postmortem MS cases, researchers found no iron rims in 67 white matter and 85 grey matter spinal cord lesions, despite most being active. In contrast, iron rims appeared in 20.9% of cortical and 80% of subcortical brain lesions, especially in deeper myelin‐rich cortical layers. These findings highlight the regional variability of iron accumulation and have important implications for interpreting iron‐rims in MS diagnosis, monitoring, and prognostication. ANN NEUROL 2026;99:730–736

The discovery of paramagnetic rim lesions (PRLs) in the multiple sclerosis (MS) brain and their association with clinical disability has enhanced our ability to diagnose, monitor, and prognosticate people living with MS. Iron's paramagnetic properties enable in vivo tracking of these “smoldering lesions” using paramagnetic‐sensitive sequences. High numbers of PRLs in the brain correlate with increased microstructural damage, global atrophy, and accumulation of irreversible disability.^1^, ^2^ Landmark magnetic resonance imaging (MRI)‐pathological studies revealed that PRLs are due to iron accumulation in microglia‐macrophages at the edge of inflammatory brain lesions.^3^, ^4^

The presence of PRLs in the spinal cord, arguably the most clinically relevant site for disability progression in MS, remains debated.^5^ Here, we provide the first systematic pathological characterization of iron‐rimmed lesions in the MS spinal cord and motor cortex. Through the study of a large autopsy cohort of progressive MS (PMS) cases, we note the conspicuous absence of histochemically detectable iron‐rimmed lesions in the spinal cord despite their presence in the brain at expected frequencies. These findings significantly advance our interpretation of iron‐rimmed lesions, and, by extension PRLs, in MS, further underscoring the need for clinically relevant biomarkers of MS spinal cord pathology.

## Materials and Methods

A well‐characterized postmortem cohort of pathologically confirmed progressive MS (n = 46) from the UK MS Tissue Bank were used (institutional review board [IRB] approval from the Imperial Research Ethics Committee, approval number: 08/MRE09/31+5). Cervical, thoracic, and lumbar cord tissue was obtained for all cases, with mesiofrontal motor cortical samples available from 36 of these MS cases.

For each case, 6 μm‐thick formalin‐fixed, paraffin‐embedded sections were labeled for non‐heme iron using an optimized DAB‐enhanced Turnbull protocol (Tirmann‐Schmeltzer Method).^6^ Adjacent sections were immunolabeled for myelin (Papanicolaou stain [PLP], BioRad), microglia‐macrophages (CD68, Dako), and ferritin (Sigma Aldrich). A subset of 20 white and 24 grey matter spinal cord lesions were immunostained for haptoglobin (Cambridge Bioscience) and ferritin (Sigma Alrich).

PLP‐ and CD68‐stained sections were used to classify lesions into active, mixed active‐inactive, and chronic inactive categories based on established criteria.^7^ Cortical lesions were additionally classified into type I to IV, as previously described.^7^, ^8^ Two independent assessors (authors M.P. and A.L.) systematically evaluated the presence of iron‐rims in demyelinated lesions and iron‐positive microglia‐macrophages.

## Results

### Population Characteristics

Cohort demographics are reported in the Table 1. A total of 136 spinal cord and 36 mesiofrontal blocks were analyzed. No demographic differences were found between the entire MS cohort (n = 46) and the subset of MS cases, wherein cortical tissue (n = 36) was also available.

**Table 1:** Population Characteristics

	Progressive Multiple Sclerosis Cohort (n = 46)		
	Mean	Range	Std. Deviation
Age at death, yr	63.50	40–92	12.96
Sex, F:M	67.4% F (31:15)		
Brain weight, g	1165.87	894–1380	132.03
Postmortem interval, h	16.989	6–31	6.76
MS symptom onset	6 progressive; 36 relapsing; 4 unknown		
Disease duration, yr	30.00	12–58	11.59
Age at wheelchair use/death, yr	51.1	28–79	13.6
Time from onset to wheelchair use/death, yr	18.97	3–50	12.03

A total of 136 spinal cord blocks from 46 progressive MS donors were systematically assessed for demyelination and iron deposition. Mesiofrontal blocks were analyzed in a subset of 36 cases, based on tissue availability. No demographic differences were found between the spinal cord cohort and the subset of MS cases wherein cortical tissue was also available.

### Iron in Spinal Cord Lesions

We identified 70 white matter lesions (active, 8/67 [11.9%]); mixed active‐inactive, (42/67 [62.7%]); and inactive (20/67 [29.9%]), and 85 grey matter lesions (active, 8/85 [9.4%]; mixed active‐inactive (40/85 [47%]); and inactive (37/85 [43.5%]).

**FIGURE 1:**
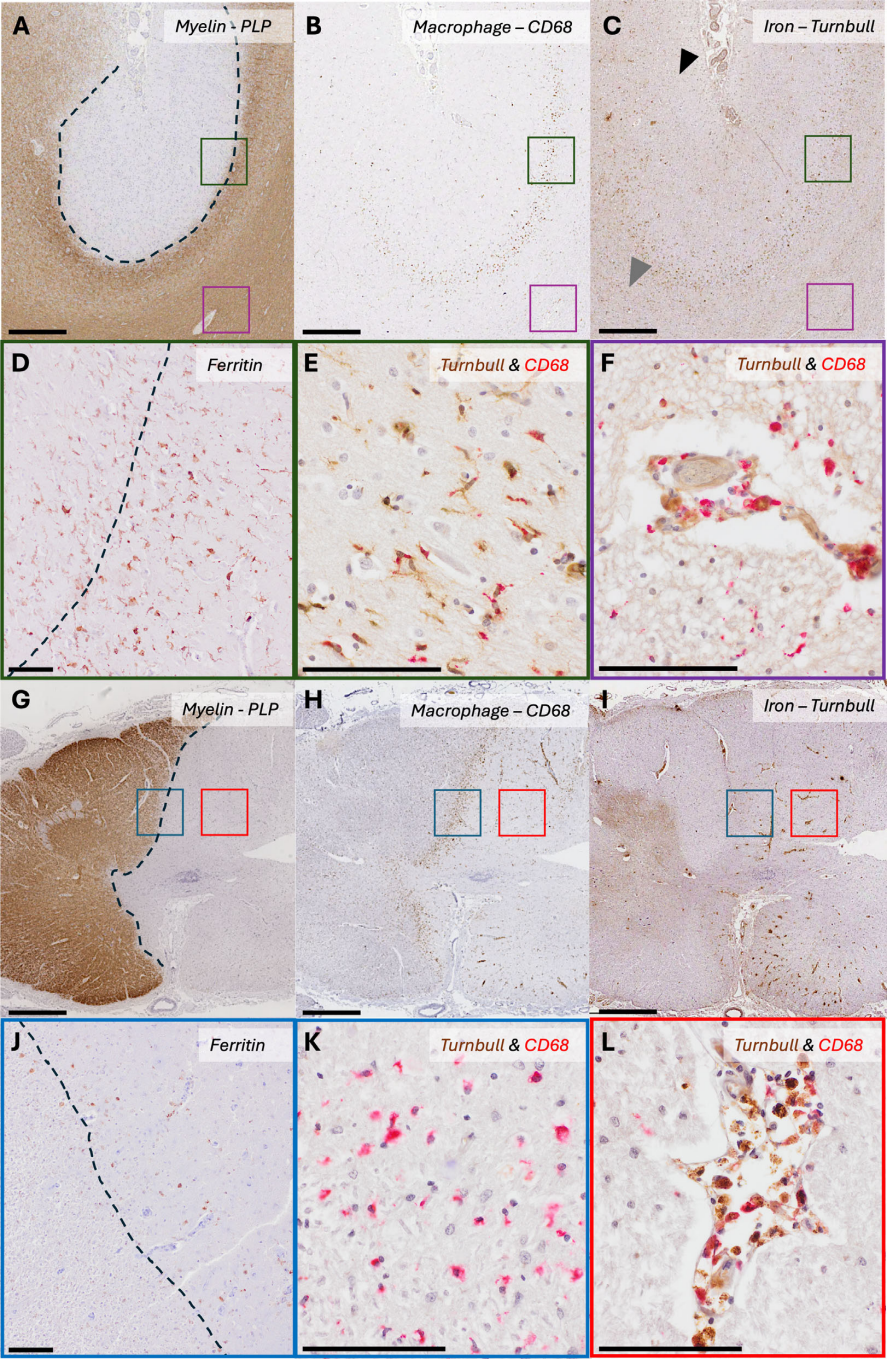
Iron‐laden macrophages accumulate at the edge of cortical and subcortical mixed active lesions but are histochemically undetectable in spinal cord lesions. (A–F) A mesiofrontal motor cortex sample from a progressive MS case with an iron‐rimmed type III cortical lesion. (A) Myelin (PLP) staining reveals a type III cortical lesion with (B) a rim of microglia‐macrophages (CD68+) at the plaque border. (C) Non‐heme iron staining using DAB‐enhanced Turnbull demonstrates reduced iron reactivity in the lesion core (*black arrowhead*), contrasting with the intense Turnbull reactivity of peri‐plaque myelin (*grey arrowhead*). A rim of iron‐positive microglia‐macrophages is visible at the plaque border. (D) The same lesion in an adjacent section displays strong ferritin immunoreactivity of macrophages at lesion edge. (E) Double labeling with Turnbull (DAB/*brown*) and the microglia‐macrophage marker CD68 (Vector Red/*red*) confirms iron reactivity in parenchymal macrophages at the plaque edge. (F) Similar double labeling highlights iron‐laden perivascular macrophages in inflammatory areas. (E, F) Abundant iron staining is observed in cortical and subcortical myelin. (G–L) A cervical spinal cord sample from a progressive MS case, showing a chronic active lesion without histochemically detectable iron accumulation. (G) Myelin (PLP) staining reveals a demyelinating plaque affecting one hemi‐cord, with (H) a rim of intense microglia‐macrophage inflammation at the plaque border. (I) Unlike cortical lesions, no iron is detected in microglia‐macrophages at the plaque rim, as shown by DAB‐enhanced Turnbull staining. (J) A faint ferritin expression is observed in some macrophages at the plaque edge. (K) Higher magnification confirms the absence of iron staining in rim macrophages, as shown by double labeling with Turnbull/DAB (*brown*) and CD68/Vector Red (*red*). (L) In contrast, iron accumulation is prominently detected in perivascular microglia‐macrophages within the plaque's edge. (K, L) Minimal iron staining is observed in spinal cord myelin compared to the brain, despite similar iron reactivity in the vasculature. The scale bar corresponds to 800 μm in A to C and G to I, and to 100 μm in D to F and J to L. MS = multiple sclerosis; PLP = Papanicolaou stain. [Color figure can be viewed at www.annalsofneurology.org]

**FIGURE 2:**
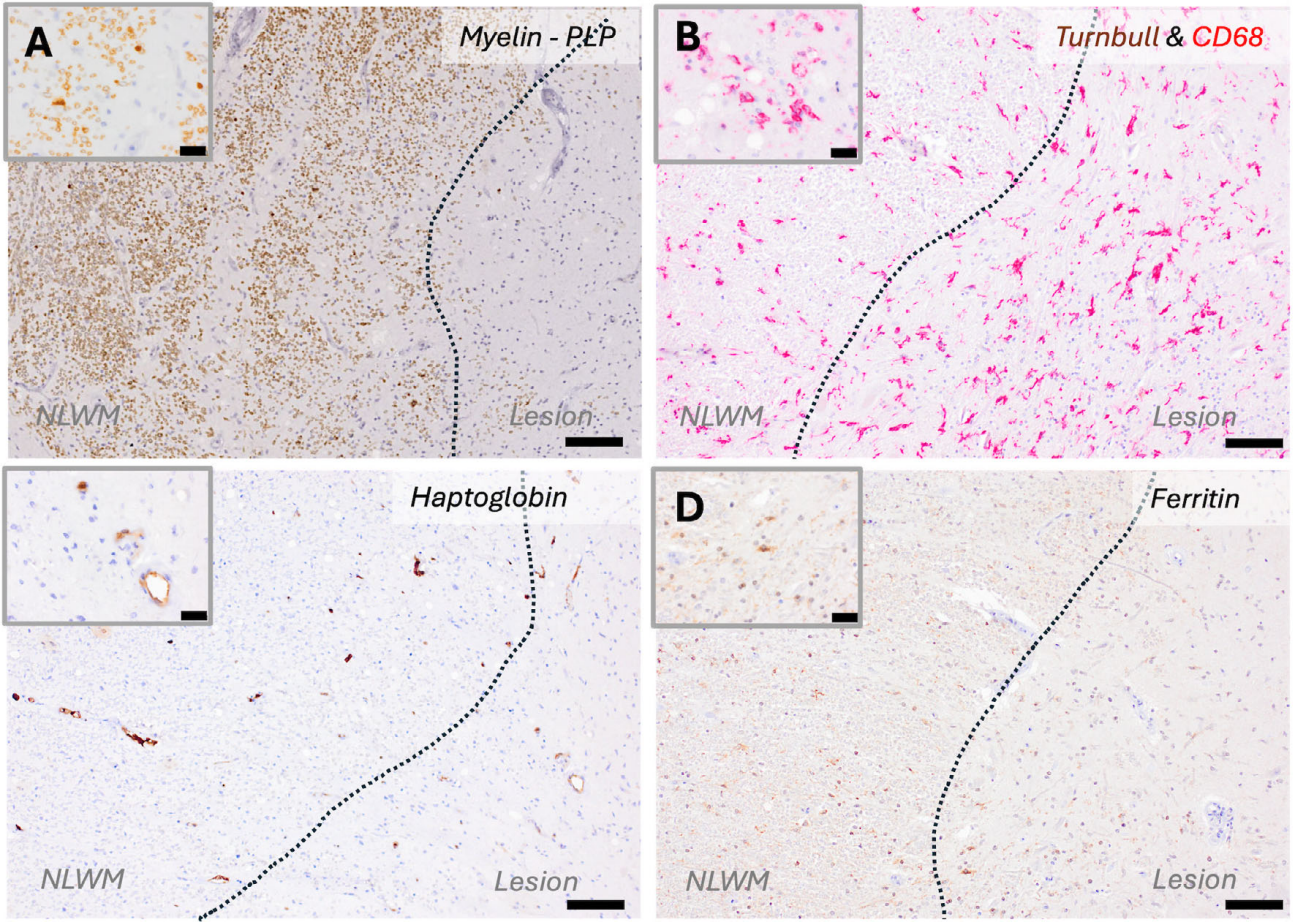
Comparative distribution of iron, ferritin and haptoglobin at the edge of a mixed active‐inactive spinal cord lesion. (A–D) The edge of a mixed active‐inactive lesion from the spinal cord of a progressive MS case. (A) Myelin (PLP) staining demonstrates the lesion edge with ongoing demyelination as demonstrated by macrophages with myelin inclusions (*inset*). (B) Double‐labeling with Turnbull/DAB (*brown*) and Iba‐1/Vector Red (*red*) shows an extensive microglia‐macrophage infiltration at the lesion edge, with several cells displaying an activated morphology (*inset*) without showing iron reactivity. (C) Haptoglobin binds free circulating hemoglobin and allows hemoglobin uptake by macrophages. Haptoglobin immunoreactivity is mostly restricted to vascular and perivascular macrophages. (D) Ferritin staining shows faint and sparse reactivity in microglia‐macrophages at the edge of the mixed active‐inactive lesion. The scale bar corresponds to 100 μm. MS = multiple sclerosis; PLP = Papanicolaou stain. [Color figure can be viewed at www.annalsofneurology.org]

None of the white matter or grey matter lesions (0/155) showed a detectable iron‐positive rim of microglia‐macrophages on Turnbull staining (Fig 1), including at the border of mixed active‐inactive lesions (0/82) where iron‐rims have been described. These findings were replicated in an additional 10 mixed active‐inactive lesions from a larger cohort.^9^ Spinal cord mixed active‐inactive lesions showed a rim of CD68‐positive microglia‐macrophages with occasional ferritin expression seen in 7 of the 44 lesions examined. Haptoglobin immunoreactivity, where present, was limited to vascular and perivascular macrophages (Fig 2). However, none of these lesions showed Turnbull reactivity (see Figs 1 and 2).

Whereas parenchymal macrophages at the edge of mixed active‐inactive lesions did not show Turnbull‐positivity, iron was found in vessel‐associated macrophages near the inflammatory lesional border (see Fig 1). Specifically, foamy macrophages with granular Turnbull‐positive cytoplasmic staining formed rich perivascular cuffs in the core of acute and mixed active lesions. In white matter lesions, perivascular iron‐laden macrophages were found in 62.5% (5/8) of active, 50% (21/42) of mixed active‐inactive, and 5% (1/20) of inactive white matter lesions. In grey matter lesions, perivascular iron‐laden macrophages were detected in 12% of active (1/8) and 2.5% of mixed active‐inactive (1/40) lesions.

Within inflammatory white matter lesions, iron‐positive axons with swollen morphology were occasionally seen, as previously described.^10^ Iron‐positive axons were observed in 2 active, and at the border of 7 mixed active‐inactive, white matter lesions. Sparse hypertrophic astrocytes had thick iron‐rich processes in gliotic cores of inactive and mixed active‐inactive white matter lesions.

Non‐lesional spinal cord tissue showed sparse and faint oligodendroglial iron staining, mostly restricted to perivascular areas.

### Iron in Cortical and Subcortical Lesions

In the MS motor cortex, we identified 43 lesions (type I, n = 6; type II, n = 5; type III, n = 21; and type IV, n = 11). Five mixed active‐inactive lesions were identified in subcortical white matter.

Turnbull staining revealed a distinct rim of Turnbull‐ and ferritin‐positivity at the border of white matter lesions with reduced iron staining in the lesion core, confirming the high iron content of subcortical myelin. Iron rimmed lesions were observed in 20.9% (9/43) of all cortical lesions studied (type I, 3/6 [50%]; type II, 0/5 [0%]; type III, 4/21 [19.1%]; and type IV, 2/11 [18.2%]; see Fig 1). Iron‐positive microglia‐macrophages were more frequently present in lesion rims involving deeper cortical layers closely situated to subcortical white matter. Iron‐laden microglia‐macrophages were found in 4 of 5 (80%) of the subcortical lesions.

Non‐lesional areas showed strong iron staining of oligodendroglia, which formed a fine fibrillar background across myelinated brain areas. The white matter showed an accentuation of iron‐reactivity along vascular trajectories which gave a typical tigroid appearance at low magnification, as previously described.^11^

## Discussion

Our study offers a detailed analysis of iron in brain and spinal cord lesions in postmortem PMS. Key findings include: (1) spinal cord lesions do not contain histochemically detectable iron‐rims; (2) cerebral cortical lesions contain iron‐rims, especially with involvement of deeper cortical layers; and (3) subcortical white matter lesions have a high prevalence of iron‐rims. Overall, our findings suggest that iron rim formation depends on microglia‐macrophages phagocytosing iron‐rich myelin during demyelination. Therefore, prevalence of PRLs likely not only depends on inflammatory state of the lesion—as established by prior histopathological studies^3^, ^4^, ^12^ but also on the regional myelin‐iron content. The topographical variation of iron accumulation in MS lesions has implications for the use of PRLs in diagnosis, monitoring, and prognosis.

Iron‐rimmed lesions in subcortical white matter strongly correlate with MS disability.^1^, ^3^ However, prior pathological studies have not examined spinal cord and cerebral cortical lesions, and MRI detection of PRLs in these regions has also been technically limited. In our study, iron was below the threshold for detection with Turnbull staining in spinal cord lesion rims, even in those which were broad with numerous activated microglia‐macrophages. Ferritin immunolabeling confirmed these histochemical findings by showing fainter and less frequent immunolabeling of microglia‐macrophages in spinal cord lesions, which contrasts with the intense ferritin immunolabeling of microglia in active rims of mesiofrontal cortical and subcortical lesions. Importantly, Turnbull iron detection in the closely situated (μm‐scale) perivascular CD68‐ and ferritin‐positive macrophages of the spinal cord suggests that our method is sufficiently sensitive to detect varying iron concentrations across the neuraxis. The difference in iron‐rich microglia/macrophages is unlikely to be solely due to reduced microglial activation in spinal cord versus brain lesions, especially as cortical grey matter lesions are typically less inflammatory (see Fig 1).^13^

The paucity of iron detected in inflammatory spinal cord lesions compared with other brain areas may explain why standard, lower resolution MRI protocols have not detected PRLs at this clinically eloquent site. To date, only one in vivo study using a 7T MRI scanner has evaluated paramagnetic signatures in the MS spinal cord.^5^ The authors described 2 lesions with increased paramagnetic signal at their borders, suggestive of PRLs. However, the authors acknowledge that these lesions bordered the subpial surface and the grey–white matter junction, regions that independently display increased paramagnetic signal in MS even in the absence of lesions. Consistent with their description, iron accumulation in subpial astrocytes and at the grey–white matter boundary in non‐lesional spinal cord tissue was also observed in our cohort (unpublished data). Nonetheless, the faint ferritin expression in macrophages at the edges of some spinal cord lesions likely suggests the presence of low concentration of iron below the detection threshold of Turnbull staining. An important caveat is that macrophages have been shown to upregulate ferritin in certain inflammatory conditions (eg, macrophage activation syndrome)^14^ even in the absence of iron. High‐field MRI with longer acquisition times may be capable of detecting PRLs in the spinal cord. Yet, regional variability in iron content must be carefully considered when interpreting the prevalence of PRLs across central nervous system (CNS) regions. Ultimately, well‐designed pathology – MRI correlation studies – will be able to resolve these discrepancies. In this context, other potential sources of a paramagnetic signal should also be taken into account.

Debate remains over whether lesion‐edge iron originates from myelin debris or the vasculature. In healthy conditions, most CNS iron is stored in myelin and oligodendrocytes, with levels highest in deep grey matter nuclei and subcortical white matter, and lowest in the spinal cord.^15^ This is confirmed by the stark contrast we observed between Turnbull reactivity in brain and spinal cord myelin. Comparing lesions from CNS regions with varying iron concentrations provides us with the unique opportunity to disentangle these 2 iron sources. We show that parenchymal microglia‐macrophage infiltration at the lesion edge decreases in iron content when transitioning from regions with high myelin iron (eg, subcortical white matter) to regions with low myelin iron (eg, cerebral cortex and spinal cord). A similar observation was made by Bagnato and colleagues, who reported that Turnbull reactivity in microglia‐macrophages in active brain lesions tended to be more intense depending on the nearby oligodendroglial iron content.^4^ Furthermore, the high iron content in the mesiofrontal cortex sampled here likely explains the higher prevalence of cortical brain lesions in our study compared with others.^16^ The gradient and topographical distribution of iron‐rims within cortical lesions, being more frequent in lesions involving infra‐granular layers with higher myelin content, further supports the association between myelin‐derived iron and PRL formation.

Alternatively, iron flux from the vasculature is supported by our observation that iron‐laden vessel‐associated macrophages were similarly found in highly inflammatory areas from both high‐ and low‐iron content myelin areas (ie, subcortical white matter and spinal cord, respectively). These cells showed reactivity for haptoglobin, a molecule that binds circulating heme iron and facilitates its uptake by macrophages via CD163. Prior studies demonstrating CD163 and heme‐oxygenase‐1 expression in microglia in brain white matter lesions also support a vascular contribution to iron accumulation in MS.^12^ Overall, our data suggest that macrophages take up iron from both myelin and circulating heme. Whereas heme iron is predominantly taken up by perivascular macrophages, myelin iron is responsible for the formation of the lesion's rim (see Fig 2). Future studies investigating iron metabolism, alongside other ionic species, in mixed active‐inactive MS lesions should be undertaken to understand better their pathogenesis and imaging correlates. Moreover, the biological underpinning of topographical differences in iron myelin content across CNS regions has not been systematically investigated to date. Regional differences in oligodendrocytes subpopulations,^17^ as well as heterogeneity of the blood–brain and blood‐spinal cord barriers,^18^ may play a role. Whether regional iron myelin content also influences the topographical heterogeneity of MS pathology, such as remyelination potential, also warrants further study.

This study reveals that despite a high prevalence of inflammatory lesions, histochemically detectable iron‐rimmed lesions are absent in the MS spinal cord, whereas they are commonly found in the brain. Our data suggest that PRL formation likely depends on both the extent of microglial/macrophage infiltration and regional myelin iron content. Therefore, PRLs likely under‐represent the full extent and nature of inflammatory activity, at least in the MS spinal cord.^15^ Future work, evaluating shifts in iron distribution and changes in its metabolism in the MS brain and spinal cord, will prove valuable in developing a clinically relevant biomarker of disease severity while also advancing our understanding of progressive MS pathogenesis.

## Author Contributions

M.P., A.L., and G.C.D. contributed to conception and design of the study; M.P., A.L., T.A., and A.A. contributed to acquisition and analysis of data; M.P., A.L., T.A., A.A, Z.D., M.B., S.H., H.D., M.H., C.L., and G.C.D. contributed to manuscript drafting and revision for intellectual content. [Correction added on 25 February 2026, after first online publication: Author contribution text has been revised in this version.]

## Potential Conflicts of Interest

Nothing to report.

## Data Availability

Raw data available upon request to the corresponding authors.
